# Preliminary Safety and Efficacy of Navitoclax Plus Ruxolitinib in Janus Kinase Inhibitor‐Naïve Patients With Myelofibrosis From the Multicenter, Open‐Label, Phase 2 Study (REFINE)

**DOI:** 10.1002/hon.70180

**Published:** 2026-03-17

**Authors:** Francesco Passamonti, James M. Foran, Anand Tandra, Valerio De Stefano, Maria Laura Fox, Ahmad Mattour, Mary Frances McMullin, Andrew C. Perkins, Gabriela Rodriguez‐Macías, Hassan A. Sibai, Akshanth R. Polepally, Yan Sun, Avijeet S. Chopra, Jason G. Harb, Qin Qin, Jalaja Potluri, Jonathan How

**Affiliations:** ^1^ Dipartimento di Oncologia ed Onco‐Ematologia Università degli Studi di Milano Fondazione IRCCS Ca’ Granda Ospedale Maggiore Policlinico Milano Italy; ^2^ Mayo Clinic Jacksonville Florida USA; ^3^ Indiana Blood and Marrow Transplant Indianapolis Indiana USA; ^4^ Section of Hematology Department of Radiological and Hematological Sciences Catholic University Rome Italy; ^5^ Fondazione Policlinico Universitario Agostino Gemelli IRCCS Rome Italy; ^6^ Department of Hematology Hospital Universitari Vall d'Hebron Experimental Hematology Vall d'Hebron Institute of Oncology (VHIO) Vall d'Hebron Hospital Campus Barcelona Spain; ^7^ Henry Ford Hospital Detroit Michigan USA; ^8^ Centre for Medical Education Queenʼs University Belfast Belfast UK; ^9^ Australian Centre for Blood Diseases Monash University and the Alfred Hospital Melbourne Australia; ^10^ Department of Hematology Hospital General Universitario Gregorio Marañón Instituto de Investigación Sanitaria Gregorio Marañón (IiSGM) Madrid Spain; ^11^ Medical Oncology and Hematology Princess Margaret Cancer Centre University of Toronto Toronto Ontario Canada; ^12^ AbbVie Inc. North Chicago Illinois USA; ^13^ Division of Hematology McGill University Health Center Montreal Quebec Canada

**Keywords:** BCL‐2, clinical trials, hematological malignancy, myelofibrosis

## Abstract

Myelofibrosis is characterized by perturbation of the JAK/STAT pathway and upregulation of anti‐apoptotic factors leading to myeloproliferation, bone marrow fibrosis (BMF), extramedullary hematopoiesis, splenomegaly, and cytopenias. Navitoclax, a potent oral B‐cell lymphoma (BCL)‐X_L_/BCL‐2 inhibitor, promotes apoptosis of malignant myelofibrosis cells. Herein, we present results of Cohort 3 of the Phase 2 REFINE study (NCT03222609), which evaluated efficacy and safety of navitoclax plus ruxolitinib in JAKi‐naïve patients with myelofibrosis. JAKi‐naïve patients with primary or secondary myelofibrosis (≥ 18 years with splenomegaly, DIPSS intermediate‐2 and high‐risk myelofibrosis, and ECOG 0–2) and platelet count > 100 × 10^9^/L were enrolled and treated with navitoclax 100 mg once daily (QD) or 200 mg QD according to platelet count (≤ 150 × 10^9^/L or > 150 × 10^9^/L, respectively). Ruxolitinib was given twice daily (dose per label). Primary endpoint: spleen volume reduction of ≥ 35% (SVR_35_) at week 24. Secondary endpoints: ≥ 50% reduction in total symptom score (TSS_50_) at week 24, change in grade of BMF, anemia response, and safety. Thirty‐two patients received ≥ 1 dose of navitoclax plus ruxolitinib. Median (range) duration of follow‐up was 44 months (5–58). 63% (20/32) of patients achieved SVR_35_ at week 24; median (range) time to first SVR_35_ was 12 weeks (11─48). Of 24 evaluable patients, 21% achieved ≥ 50% reduction in driver gene variant allele frequency (VAF). Of 27 evaluable patients, 11 (41%) achieved TSS_50_ at week 24; median (range) time to first TSS_50_ of 3 weeks (0─16). BMF improved from baseline by ≥ 1 grade in 13/27 patients (48%) at any time on study. Anemia response rates were 38% (5/13) for transfusion‐independent and 100% (2/2) for transfusion‐dependent patients. No bleeding events or deaths were attributed to navitoclax. These findings suggest navitoclax plus ruxolitinib has a tolerable safety profile and provides clinically meaningful improvements for JAKi‐naïve patients with myelofibrosis.

**Trial Registration:**

NCT03222609.

## 1 Introduction

Myelofibrosis is a rare hematologic malignancy, typically presenting in people > 60 years old [1]. Clinical manifestations include anemia, splenomegaly, thrombocytopenia, constitutional symptoms and other symptoms like fatigue, which significantly impair quality of life (QoL) and reduce life expectancy. Median overall survival (OS) is > 10 years in low‐risk, < 5 for intermediate‐2 risk, and < 2 years for high‐risk patients [2, 3].

Myelofibrosis features recurrent mutations in genes such as Janus kinase 2 (*JAK2*) V617F (the most frequent driver mutation), calreticulin (*CALR*), and *MPL* [2, 4]. These mutations constitutively activate the JAK/STAT pathway, leading to upregulation of anti‐apoptotic factors like B‐cell lymphoma (BCL)‐X_L_ and myeloid cell leukemia sequence 1 (MCL‐1), promoting increased cell proliferation and survival [5, 6, 7, 8]. The JAK inhibitor (JAKi) ruxolitinib is currently first‐line therapy for intermediate‐ or high‐risk myelofibrosis [9]. Long‐term studies show ruxolitinib can reduce spleen volume and improve symptomatology, with some data demonstrating prolonged OS [1, 10, 11, 12, 13]. However, JAKi monotherapy has not demonstrated true disease modification, and less than half of the patients have optimal response, typically lasting only 1–3 years [11, 13, 14, 15].

Navitoclax is an oral small‐molecule BCL‐2 homology 3 (BH3) mimetic that binds with high affinity to anti‐apoptotic proteins BCL‐X_L_ and BCL‐2, promoting apoptosis of malignant cells [16]. In preclinical studies, navitoclax combined with ruxolitinib shows synergy in inducing malignant cell death, with ruxolitinib inhibiting upstream *JAK2* signaling, while navitoclax provides downstream inhibition of survival factors [5, 17, 18]. Combining navitoclax with ruxolitinib in patients with progression or suboptimal response to ruxolitinib monotherapy may modify underlying disease [19, 20], with durable reduction in splenomegaly and bone marrow fibrosis (BMF), improved symptoms, and anemia response [20].

Although ruxolitinib monotherapy represents the therapeutic backbone in myelofibrosis, it does not alter the underlying disease [15]. Herein, we present results from Cohort 3 of the REFINE Phase 2 study, evaluating the efficacy and safety of navitoclax plus ruxolitinib in JAKi‐naïve patients with myelofibrosis.

## 2 Methods

### 2.1 Study Design, Patients, and Treatment

Cohort 3 of the Phase 2 multicenter REFINE study (NCT03222609) enrolled JAKi treatment‐naïve patients aged ≥ 18 years with primary or secondary myelofibrosis. Inclusion and exclusion criteria are located within the Supporting Information S1.

**FIGURE 1:**
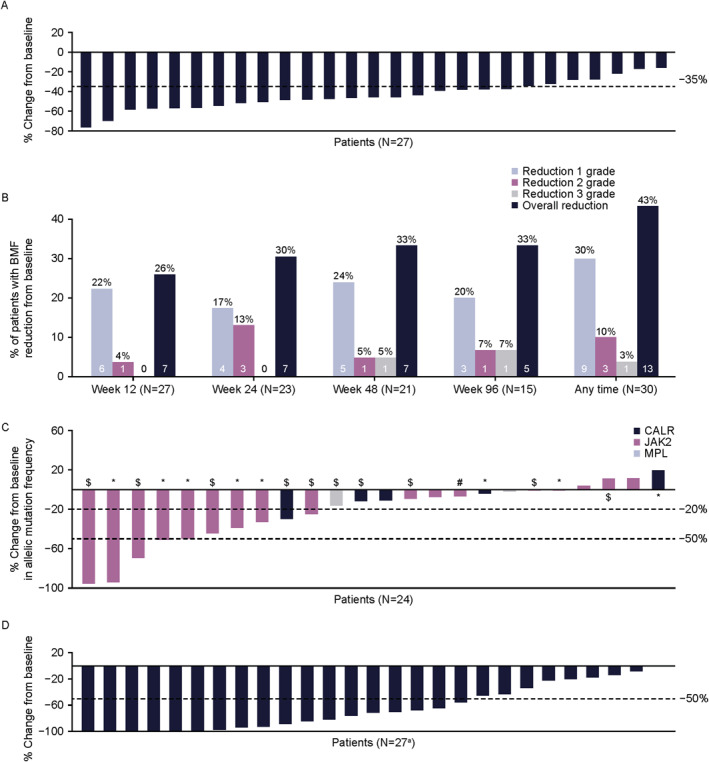
Efficacy responses over time. (A) Percentage change from baseline in SVR at week 24 (B) percentage of patients with BMF reduction from baseline over time (C) percentage change from baseline in allelic mutation frequency at week 24 (D) percentage change from baseline in TSS_50_ at any time during the study treatment. ^a^One patient had 0% change from baseline in TSS_**50**_ at any time during the study treatment. *Achieved SVR_35_ and TSS_50_ at week 24; $ achieved SVR_35_ at week 24 only; # achieved TSS_50_ at week 24 only. BMF, bone marrow fibrosis; SVR, spleen volume reduction; TSS_50_, ≥ 50% reduction in total symptom score.

Patients initiated treatment with navitoclax 100 mg once daily (QD) if baseline platelet count was ≤ 150 × 10^9^/L, or 200 mg QD if baseline platelet count was > 150 × 10^9^/L (Supporting Information S1: Figure 1). For patients initiating treatment with navitoclax 100 mg, the dose could be increased to 200 mg after 7 days once platelet count remained ≥ 75 × 10^9^/L. The dose could be increased to 300 mg QD in patients with suboptimal spleen response (failure to reach spleen volume reduction [SVR] > 10%) following week 24 disease assessment. Ruxolitinib was given twice daily with starting dose based on baseline platelet count per local label [21].

The study was approved by local ethics committees (protocol number: M16‐109) and conducted in accordance with the International Conference on Harmonization, Good Clinical Practice guidelines, and the Declaration of Helsinki. All patients provided written informed consent.

### 2.2 Endpoints and Assessments

The primary efficacy endpoint was SVR_35_ from baseline at week 24, measured by magnetic resonance imaging or computer tomography according to International Working Group criteria [22]. The analysis window for week 24 was defined as days 127–211 per study activity schedule.

Secondary efficacy endpoints were: (i) ≥ 50% reduction in total symptom score (TSS_50_) from baseline at week 24 as assessed by the MFSAF v4.0 [23, 24]; (ii) change in grade of BMF, assessed locally per European consensus grading system [25]; (iii) anemia response (hemoglobin improvement of ≥ 2 g/dL and transfusion independence) per International Working Group for Myeloproliferative Neoplasms Research and Treatment [22, 26]; (iv) to describe the safety and pharmacokinetics (PK) profile observed with the navitoclax and ruxolitinib combination. Blood samples were collected for PK analysis of navitoclax and ruxolitinib trough concentrations up to week 24 (days 1–169) and concentrations at 4 h post‐dose on day 1. Key exploratory endpoints and detailed information about statistical analyses are available in the Supporting Information S1.

Safety assessments included duration of exposure, adverse events (AEs), serious AEs (SAEs), and deaths, and were graded according to National Cancer Institute common terminology criteria for adverse events (NCI CTCAE) v4.03 [27].

## 3 Results

### 3.1 Patient Demographics and Baseline Characteristics

**FIGURE 2:**
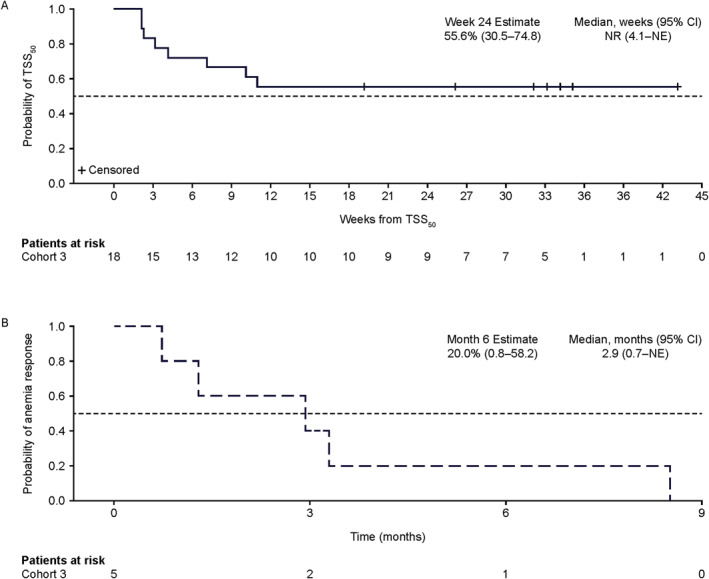
Probability of treatment outcomes over time. (A) Durability of TSS_50_ over time (B) duration of anemia response. CI, confidence interval; NE, not estimable; TSS_50_, ≥ 50% reduction in total symptom score.

At data cutoff, March 03, 2025, 32 patients with myelofibrosis received ≥ 1 navitoclax plus ruxolitinib dose. The median (range) age was 69 years (44–83), and 63% were male (*n* = 20); median (range) spleen volume was 1889 cm^3^ (646–7340), and 40% (*n* = 13) had ≥ 1 prior line of therapy for myelofibrosis (Table 1). DIPSS risk at study entry was low for 1 (3%), intermediate‐1 for 28% (*n* = 9), intermediate‐2 for 59% (*n* = 19), and high for 9% (*n* = 3); low‐risk patient was included due to incorrect interpretation of cytogenetics and overall risk categorization by DIPSS. Twenty‐eight (88%) patients received a navitoclax 200 mg QD starting dose, and 4 (13%) received 100 mg QD. Baseline median (range) VAF was 56.5% (0%–96%), with 22 (69%) having *JAK2*, 6 (19%) having *CALR*, 3 (9%) having *MPL* mutations, and 1 (3%) patient being triple‐negative for *JAK2*, *CALR*, and *MPL*. Additionally, 19 (59%) patients had high molecular‐risk (HMR) mutations (e.g., *ASXL1*, *SRSF2*, *EZH2*, *U2AF1 (Q157)*, *IDH1* or *IDH2*; Supporting Information S1: Figure 2).

**TABLE 1:** Baseline demographics and disease characteristics.

Characteristics	Overall (*N* = 32)
Age, median (range), years	69 (44─83)
Time from diagnosis of MF to study entry, months, median (range)	16.7 (0.7─247.5)
Baseline spleen volume, cm^3^, median (range)	1889 (646─7340)
Gender	
Female	12 (38)
Male	20 (63)
Race	
White	29 (91)
Black or african american	2 (6)
Multiple	1 (3)
Asian	0
Ethnicity	
Hispanic or latino	6 (19)
Not hispanic or latino	26 (81)
ECOG performance status	
0	13 (41)
1	17 (53)
2	2 (6)
Risk group by DIPSS at study entry	
Low	1 (3)
Intermediate‐1	9 (28)
Intermediate‐2	19 (59)
High	3 (9)
Bone marrow fibrosis grade^a^	
Grade 0–1	0
Grade 2–3	30 (94)
Missing	2
TSS, median (range)	12.0 (1.0─50.4)
Hemoglobin level, g/dL, median (range)	10.3 (4.4─13.7)
Hemoglobin level < 10 g/dL	15 (49)
Transfusion status	
Dependent	2 (6)
Independent	30 (94)
Baseline VAF, median (range), %	56.5 (0–96)
Presence of driver mutations	
*JAK2*	22 (69)
*CALR*	6 (19)
*MPL*	3 (9)
Triple‐negative *(JAK2, CALR, MPL)*	1 (3)
Presence of high molecular‐risk mutations^b^	19 (59)
*ASXL1*	14 (44)
*SRSF2*	4 (13)
*EZH2*	4 (13)
*U2AF1*	1 (3)
*IDH2*	1 (3)

*Note:* Data are *n* (%) unless otherwise stated.

Abbreviations: DIPSS, dynamic international prognostic scoring system; ECOG, eastern cooperative oncology group; MF, myelofibrosis; TSS, total symptom score.

^a^ 2 Patients had missing baseline values; percentages were calculated using nonmissing values.

^b^ Defined as mutations in *ASXL1*, *SRSF2*, *EZH2*, *IDH1/2*, or *U2AF1*.

**FIGURE 3:**
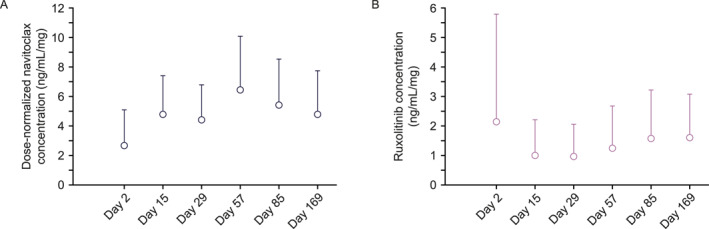
Pre‐dose dose‐normalized plasma concentrations during scheduled cycle visits. (A) Navitoclax plasma concentrations (B) ruxolitinib plasma concentrations.

All 32 patients discontinued both navitoclax and ruxolitinib. For navitoclax, 6 discontinuations were due to AEs (2 thrombocytopenia, 2 atrial fibrillation, 1 neutropenia, and 1 cardiac disorder) and 3 due to progressive disease; and for ruxolitinib, 4 discontinuations were due to AEs (2 thrombocytopenia, 1 atrial fibrillation, and 1 cardiac disorder) and 3 due to progressive disease. All reasons for study drug discontinuation are shown in Supporting Information S1: Figure 3. Study discontinuation was reported for all 32 patients (100%); 2 patients withdrew consent, 10 deaths were reported, and 20 patients discontinued the study for other reasons (18 discontinued due to the sponsor terminating the study and 2 discontinued due to becoming eligible and/or pursuing transplant). The median (range) duration of follow‐up was 44 months (5–58) months.

### 3.2 Efficacy Assessments

#### 3.2.1 Spleen Volume Reduction

SVR_35_ was achieved in 20 (63%) patients at week 24 (Figure 1A) and in 26 (81%) at any time during the study; these patients (*n* = 26) had an observed median duration of SVR_35_ of 19 months. Estimated median duration of SVR_35_ in the total population was not yet reached (Table 2). The median (range) time to first SVR_35_ was 12 (11─48) weeks. Within subgroups known to confer poor prognosis, SVR_35_ was achieved by 74% (*n* = 17/23) aged ≥ 65 years, 74% (*n* = 14/19) with intermediate‐2 DIPSS score, 100% (*n* = 3/3) with high DIPSS score, and 68% (*n* = 13/19) with HMR mutations at any time during study treatment. Median (range) duration of SVR_35_ achieved at any time on study was 15 (0–31) months in 26 evaluable patients. A reduction of ≥ 50% in palpable splenomegaly from baseline was achieved in 19/25 (76%) evaluable patients at week 24 and 22/27 (81%) at any time on study. The majority (28/32) received 200 mg as a starting dose of navitoclax based on their baseline platelet count of > 150 × 10^9^/L. Among these patients, SVR_35_ responders (*n* = 18) had a higher median average navitoclax dose of ∼130 mg compared with ∼100 mg for non‐responders (*n* = 10) over 24 weeks of treatment.

**TABLE 2:** Summary of response assessments.

Assessment	Overall (*N* = 32)
SVR_35_	
Week 24	20 (63)
Any time	26 (81)
Duration of SVR_35_, median months (range)^a^	
All patients (observed)	18.8 (0.1–47.9)
All patients (estimated)	NE (19.3—NE)
TSS_50,_	
Week 24	11 (34)
Any time	18 (56)
BMF improvement by ≥ 1 grade	
Week 24	7/23 (30)
Any time	13/27 (48)
Anemia response	7/15 (47)

*Note:* Data are *n* (%) unless otherwise stated.

Abbreviations: BMF, bone marrow fibrosis; NE, not estimable; SVR_35_, spleen volume reduction of ≥ 35%; TSS_50_, ≥ 50% reduction in total symptom score.

^a^ Data missing for *n* = 6.

#### 3.2.2 Bone Marrow Fibrosis

Of 27 patients with matched baseline and post‐baseline results (2 had missing baseline grade and 3 had missing post‐baseline grade), BMF improved from baseline by ≥ 1 grade in 13/27 (48%) at any time on study (95% CI: 29–68): 7/27 (26%) at week 12, 7/23 (30%) at week 24, 7/21 (33%) at week 48, and 2/7 (29%) at week 96 (Figure 1B and Table 2). Of 12/27 patients with improved BMF, 8 improved by 1 grade and 4 by 2 grades; 3 had BMF reductions at all 3 visits (weeks 12, 24, and 48). Of the remaining patients, 13/27 had equal BMF grades (10 of whom were grade 3 at baseline) and 2/27 had worsened BMF grades, with 22/27 having grade 3 fibrosis at baseline. Of the 13 patients who had reduction in BMF, 11 (85%; 95% CI: 55─98) experienced SVR_35_ at any time on study.

### 3.3 Biomarker Data

Of 24 evaluable patients at week 24 with *JAK2, CALR*, or *MPL* mutations, 10 (42%; 95% CI: 22─63) achieved ≥ 20% reduction in allele frequency from baseline; 5/14 (36%) were in the HMR group, 5/10 (50%) were in the non‐HMR group, and 5 (21%) achieved ≥ 50% reduction in driver gene variant allele fraction (VAF) (Figure 1C). Of 31 evaluable patients, 15 (48%) and 6 (19%) achieved ≥ 20% and ≥ 50% reduction in *CALR*, *JAK2*, or *MPL* allele frequency at any time during study, respectively.

### 3.4 Reduction in Total Symptom Score

Of 32 evaluable patients, 11 (34%) achieved TSS_50_ at week 24 and 18 (56%) at any time during study treatment (Figure 1D and Table 2). Median (range) time to first TSS_50_ was 3.2 weeks (0.3─16.3). Median duration of TSS_50_ was not yet reached (Figure 2A). Of 23 patients with matched baseline and postbaseline data, mean (SD) TSS change from baseline was −9.4 (15.8). A meaningful change threshold (MCT) for TSS of at least 7 points was derived using anchor‐based analyses. Based on this MCT, clinically meaningful change was observed in 11 patients (34%) at week 24 and 12 patients (38%; 95% CI: 21─56) at any time on study (data on file).

### 3.5 Other Patient‐Reported Outcomes

Of 22 evaluable patients, mean (SD) change in fatigue from baseline at week 24 was −0.8 (0.8) points (95% CI: −1.1 to −0.4), as measured by the PROMIS Short Form v1.0—Fatigue 7a; the decrease in symptom score was maintained until week 156. Mean (SD) change in patient Global Health Status/QoL from baseline among the 26 evaluable patients at week 24 was 15.4 (24.4) points (95% CI: 5.6–25.2), as measured by the EORTC QLQ‐C30, and including fatigue, insomnia, pain, appetite loss, dyspnea, financial difficulties, diarrhea, constipation, and nausea and vomiting; increases in scores were maintained until Week 180. The mean (SD) change from baseline at week 24, as measured by the EORTC QLQ‐C30, was −18.1 (25.1) points (95% CI: −28.0 to −8.2) for fatigue and −13.0 (27.5) points (95% CI: −23.8–−2.1) for pain in 27 evaluable patients, and −16.7 (34.3) points (95% CI: −30.5 to −2.8) for insomnia in 26 evaluable patients.

### 3.6 Anemia Responses

Of 13 patients who were transfusion‐independent with hemoglobin < 10 g/dL at baseline, 5 (38%) had an anemia response (hemoglobin improvement of ≥ 2 g/dL). Both patients who were transfusion‐dependent at baseline (2/2) achieved transfusion independence in response to therapy. Among responders who were transfusion independent with baseline hemoglobin < 10 g/dL, estimated median (95% CI) duration of anemia response achieved at any time on study was 3 (0.7–not estimable [NE]) months (Figure 2B).

### 3.7 Time‐to‐Event Endpoints

Median OS was NE (95% CI: 41 months–NE). With a median (range) follow‐up of 44 months (5–58), the estimated OS at 24 months is 80% (95% CI: 61–91). Median PFS was 41 months (95% CI: 23–NE). The estimated PFS at 24 months is 65% (95% CI: 38–82).

### 3.8 Pharmacokinetics

The dose‐normalized mean pre‐dose concentrations of navitoclax ranged between 2.6 ng/mL/mg and 6.4 ng/mL/mg and ruxolitinib ranged between 0.95 ng/mL/mg and 2.1 ng/mL/mg across visits (day 2 to day 169) (Figure 3). The dose‐normalized mean 4‐h post‐dose concentrations for navitoclax and ruxolitinib were 7.0 ng/mL/mg and 5.8 ng/mL/mg, respectively.

### 3.9 Safety

Since study initiation, median (range) exposure to both navitoclax and ruxolitinib was 126 (1–220) weeks. Overall, 26 (81%) reached a maximum navitoclax dose of 200 mg QD and 4 (13%) escalated to maximum 100 mg QD. Median dose intensity of navitoclax was 101 mg/d, and median dose intensity of ruxolitinib was 20 mg/d. All patients who remained on navitoclax at week 24 received ≥ 50 mg dose. Navitoclax dose reduction due to AEs occurred in 26 (81%) patients; dose interruptions occurred in 20 (63%) and discontinuations occurred in 6 (20%). Interruption mostly occurred due to thrombocytopenia in 12 (38%) patients (*n* = 11 with grade 3). The patients who experienced navitoclax dose interruption restarted at the same or lower dose within a couple weeks. The most common navitoclax dose after reduction from 200 mg was 100 mg (Supporting Information S1: Figure 4). Ruxolitinib dose reductions due to AEs occurred in 17 (53%) patients; interruptions in 17 (53%), and discontinuations in 4 (12%).

All 32 (100%) patients experienced ≥ 1 AE, 28 (88%) had grade ≥ 3 AEs, and 12 (38%) had SAEs (Table 3). The most common any‐grade AEs were anemia (*n* = 21, 66%), thrombocytopenia (*n* = 18, 56%), diarrhea (*n* = 18, 56%), and COVID‐19 infection (*n* = 12, 38%). The most common grade ≥ 3 AEs were thrombocytopenia (*n* = 12, 38%) without clinically significant bleedings, anemia (*n* = 12, 38%), and neutropenia (*n* = 8, 25%). There were no SAEs of interest, and atrial fibrillation (*n* = 2) was the only SAE to occur in > 1 patient. Thrombocytopenia was manageable and reversible on dose reduction/interruption of navitoclax or ruxolitinib. Median (range) time to first thrombocytopenia event was 21 (1–450) days, and the median time to initial onset of first thrombocytopenia that eventually led to navitoclax dose interruption was 29 (2–1172) days. No significant trends were observed in hematologic parameters over time (Supporting Information S1: Figure 5). The mean platelet count was ≥ 100 × 10^9^/L at 8, 12, and 24 weeks after initiation of combined treatment (Supporting Information S1: Figure 5). No bleeding events occurred with co‐existing thrombocytopenia, and none were attributed to navitoclax. Of the 10 (31%) patients who died, 3 (9%) died ≤ 30 days after the last dose of navitoclax; no deaths were deemed related to navitoclax or ruxolitinib.

**TABLE 3:** Overview of safety.

	Overall (*N* = 32)
Any AE	32 (100)
Any AE related to navitoclax	32 (100)
Any AE related to ruxolitinib	30 (94)
Grade 3 or 4 AEs	28 (88)
AE leading to discontinuation of navitoclax	6 (19)
AE leading to discontinuation of ruxolitinib	4 (13)
AE leading to interruption of navitoclax	20 (63)
AE leading to interruption of ruxolitinib	17 (53)
AE leading to reduction of navitoclax	26 (81)
AE leading to reduction of ruxolitinib	17 (53)
All deaths^a^	10 (31)
Deaths ≤ 30 days after last navitoclax dose	3 (9)
Deaths > 30 days after last navitoclax dose	7 (22)
Most common any‐grade AEs (occurring in ≥ 20% of patients)	32 (100)
Anemia	21 (66)
Thrombocytopenia	18 (56)
Diarrhea	18 (56)
COVID‐19 infection	12 (38)
Neutropenia	10 (31)
Nausea	10 (31)
Fatigue	9 (28)
Most common grade ≥ 3 AEs (occurring in ≥ 10% of patients)	28 (88)
Thrombocytopenia	12 (38)
Anemia	12 (38)
Neutropenia	8 (25)
Serious AEs	12 (38)

*Note:* Data are *n* (%) unless otherwise specified.

Abbreviation: AE, adverse event.

^a^ Includes non‐treatment–emergent deaths. Treatment‐emergent causes of death include myocardial infarction and cardiac arrest (*n* = 1; 3%), general disorders and administration site conditions (*n* = 1; 3%), and respiratory failure (*n* = 1; 3%).

## 4 Conclusions

In this Phase 2 study, treatment with navitoclax and ruxolitinib demonstrated manageable safety and clinical benefit with preliminary evidence of disease modification among JAKi‐naïve patients with myelofibrosis. All patients experienced reduction in spleen volumes during study treatment. The primary endpoint, SVR_35_, was achieved by 63% of patients at week 24, with a median duration of SVR_35_ of 15 months. This observed rate of ≥ 35% reduction in spleen volume is historically comparable to the 63% of patients that achieved SVR_35_ at week 24 for JAKi‐naïve patients with myelofibrosis treated with navitoclax plus ruxolitinib in the Phase 3 TRANSFORM‐1 trial, and appears to be represent an improvement over data within the same study showing 32% of patients achieving SVR_35_ for placebo‐controlled patients treated with ruxolitinib alone [28].

Though data are limited, patients with a higher median average navitoclax dose (∼130 vs. ∼100 mg) and no dose adjustments throughout the study had higher SVR_35_ rates. Additionally, dose reductions due to AEs occurred in 81% of patients, and dose interruptions from thrombocytopenia in 38%, resulting in a median navitoclax dose intensity of 101 mg/day—below the intended maximum of 200 mg/day. While objective responses occurred across various doses, suggesting target inhibition does not require the maximum dose for all patients, these data indicate the therapeutic window may be limited by thrombocytopenia and that individualized dosing strategies may be needed to optimize outcomes.

The approved JAKis ruxolitinib, fedratinib, pacritinib, and momelotinib have little/no consistent impact on BMF, VAF, leukemic transformation or OS, highlighting the unmet need for novel therapies with disease‐modifying activity [3, 29, 30, 31, 32, 33]. Frontline treatment with navitoclax and ruxolitinib resulted in reduction of BMF as early as week 12 in 7/27 (26%) patients, which compares favorably to longer‐term observations that have reported reductions in fibrosis at last follow‐up (median 2.2 years) for 16% of patients treated with ruxolitinib alone [11]. More than 80% of patients with reduction in BMF also achieved SVR_35_.

Approximately 20% of evaluable patients obtained ≥ 50% reduction in driver gene mutations, some as early as week 12. Notably, 53% of patients with JAK2V617F at baseline had ≥ 20% reduction in VAF for *JAK2* ^*V617F*^ at week 24. This decrease was observed at an earlier timepoint and for a greater numerical proportion than what was observed in the COMFORT‐II trial at week 168 and week 192, with 38% and 31% of evaluable *JAK2* ^*V617F*^‐positive patients treated with ruxolitinib monotherapy showing *a* > 20% reduction from baseline in absolute allele burden [11]. Slower kinetics in the reduction of *JAK2* ^*V617F*^ allele burden with ruxolitinib were also observed in longer‐term finding up to 216 weeks [34], highlighting the potential disease‐modifying activity of navitoclax. Although at diagnosis, a high *JAK2* ^*V617F*^ burden (vs. lower) in myelofibrosis has been previously associated with better OS [35, 36], in this study, lower *JAK2* ^*V617F*^ burden was associated with clinical responses, as 9‐out‐of‐9 patients that achieved *a* ≥ 20% reduction by week 24 also achieved TSS_50_ and/or SVR_35_ at week 24.

Administration of combination therapy also had a positive effect on patient‐reported outcomes. While the proportion of patients that achieved TSS_50_ at week 24 was numerically lower for patients treated with navitoclax plus ruxolitinib compared with historical values for ruxolitinib alone (34% vs. 46%) [37], almost 60% of evaluable patients achieved TSS_50_ at any time during the study. These data suggest that although responses may be delayed or variable in timing with the addition of navitoclax, most patients do see an improvement in their symptom response. Additionally, patients reported improvement in both fatigue signs/symptoms and impact of fatigue, as well as overall improvement in their QoL, with reduction in fatigue, pain, and insomnia symptoms. Nonetheless, treatment‐related AEs, especially fatigue (28%) and diarrhea (56%), may have partially offset disease symptom relief, as these symptoms contribute to the TSS. Their emergence or worsening can mask net improvement, particularly early in therapy (pre‐week 24) when patients are adjusting and AE burden is high. Thus, TSS_50_ may underrepresent the true benefit of treatment in the presence of on‐treatment toxicities.

Thrombocytopenia and anemia were the most frequent any‐grade and grade ≥ 3 hematologic AEs. As BCL‐X_L_ is essential for platelet survival, higher incidences of thrombocytopenia were expected with navitoclax. Although thrombocytopenia rates with navitoclax plus ruxolitinib were higher than ruxolitinib alone [11, 37], all cytopenias were reversible and manageable with dose modification, and no bleeding events or deaths were attributed to navitoclax.

Study limitations include the open‐label study design, lack of a comparator arm, and small sample size, which limits definitive conclusions regarding efficacy. In particular, the study population largely consisted of patients with mild baseline anemia (40.6% had hemoglobin < 10 g/dL; 2 were transfusion‐dependent). This may limit the generalizability of the anemia response findings to those with more severe anemia or frequent transfusions.

The navitoclax and ruxolitinib combination reduced splenomegaly and symptoms, which are prominent manifestations of myelofibrosis, and provided some evidence for potential disease modification through reduction in BMF and clonal burden. Toxicity was mostly managed with dose modification. These promising findings for navitoclax plus ruxolitinib in JAKi treatment‐naïve patients with myelofibrosis provide valuable insight into the potential role of navitoclax in this population and inform on future clinical development and identification of patient populations most likely to benefit from novel therapies, including ongoing studies of navitoclax in other hematologic malignancies, such as acute myeloid leukemia (NCT05222984) and myelodysplastic syndromes (NCT05564650). Results from the ongoing Phase 3 placebo‐controlled trial (TRANSFORM‐1, NCT04472598) will support these observations.

## Author Contributions

Conceptualization: Jalaja Potluri, Akshanth R. Polepally, Avijeet S. Chopra, Jason G. Harb, Qin Qin. Formal analysis: Yan Sun, Jason G. Harb, Qin Qin. Investigation: Francesco Passamonti, James Foran, Anand Tandra, Valerio De Stefano, Maria Laura Fox, Ahmad Mattour, Mary Frances McMullin, Andrew C. Perkins, Gabriela Rodriguez‐Macías, Hassan A. Sibai, Jonathan How. Project administration: Francesco Passamonti, James Foran, Anand Tandra, Valerio De Stefano, Maria Laura Fox, Ahmad Mattour, Mary Frances McMullin, Andrew C. Perkins, Gabriela Rodriguez‐Macías, Hassan A. Sibai, Jonathan How, Avijeet S. Chopra, Qin Qin, Jalaja Potluri. Writing – Review and Editing: All authors.

## Funding

AbbVie funded this study and participated in the study design, research, analysis, data collection, interpretation of data, reviewing, and approval of the publication. All authors had access to relevant data and participated in the drafting, review, and approval of this publication. No honoraria or payments were made for authorship.

## Ethics Statement

The study was approved by local ethics committees (protocol number: M16‐109) and conducted in accordance with the International Conference on Harmonization, Good Clinical Practice guidelines, and the Declaration of Helsinki.

## Consent

All patients provided written informed consent.

## Conflicts of Interest

F. Passamonti: Received honoraria during the last 2 years for lectures from Novartis, Bristol‐Myers Squibb, Abbvie, GSK, AOP Orphan, Jazz, Menarini Stemline and for advisory boards from Novartis, Bristol‐Myers Squibb/Celgene, GSK, Abbvie, Keros, Sumitomo. J.M. Foran: Formal advisory activities: Novartis, Servier, Pfizer, BMS, Taiho, Research support (Institution): AbbVie, Actinium, Aptose, Astex, H3Biosciences, Kura Oncology, Trillium, Xencor. A. Tandra: Received honoraria from Gilead, ADC Therapeutics, Alexion, and Sanofi‐Genzyme. V. De Stefano: Received honoraria from AbbVie, AOP Orphan Pharmaceutical, Novartis, and BMS. M.L. Fox: Honoraria and advisory board for Novartis, Takeda, GSK, AbbVie, and BMS. A. Mattour: Consultancy: GSK; Stock: AbbVie, Amgen, BMS, KTRA, J&J. M.F. McMullin: Consultancy: Novartis, BMS, GSK; Research funding: BMS, AOP. A.C. Perkins: Honoraria and advisory boards: Novartis Oncology, AbbVie, GSK, CTI, BMS, Kartos. G. Rodriguez‐Macías: Advisory role ‐ Jazz, Novartis, BMS‐Celgene, AbbVie, Servier, Astellas, Otsuka. H.A. Sibai: Honoraria for consulting or advisory role: Novartis, BMS, Gilead Sciences, AbbVie, Jazz, Pfizer. A.R. Polepally, Q. Qin, Y. Sun, J.G. Harb, A.S. Chopra, J. Potluri: Employees of AbbVie and may own stock. J. How: Honoraria: Novartis, Teva, Astellas, GSK; Consultancy: Novartis, Ionis; Speakers Bureau: Novartis, Astellas; Research Funding: AbbVie, Astellas, Sierra Oncology; Travel expenses: Jazz Pharmaceuticals.

## Supporting information


Supporting Information S1

## Data Availability

AbbVie is committed to responsible data sharing regarding the clinical trials we sponsor. This includes access to anonymized, individual, and trial‐level data (analysis data sets), as well as other information (e.g., protocols, clinical study reports, synopses, or statistical analysis plans), as long as the trials are not part of an ongoing or planned regulatory submission. These clinical trial data can be requested by any qualified researchers who engage in rigorous, independent, scientific research, and will be provided following review and approval of a research proposal, Statistical Analysis Plan (SAP), and execution of a Data Use Agreement (DUA). Data requests can be submitted at any time after approval in the US and Europe and after acceptance of this manuscript for publication. The data will be accessible for 12 months, with possible extensions considered. For more information on the process or to submit a request, visit the following link: https://vivli.org/ourmember/abbvie/ then select “Home”.
