# Supplementary material for: Preliminary Safety and Efficacy of Navitoclax Plus Ruxolitinib in Janus Kinase Inhibitor‐Naïve Patients With Myelofibrosis From the Multicenter, Open‐Label, Phase 2 Study (REFINE)
Source: Hematol Oncol. 2026 Mar 17;44(2):e70180. doi: 10.1002/hon.70180 (PMC12996732; doi:10.1002/hon.70180)
Supplement: Supplementary file 1 — Supporting Information S1 [file HON-44-e70180-s001.docx]

# Supplementary material

**Supplemental methods**

**Inclusion and exclusion criteria.** Patients had splenomegaly, Dynamic International Prognostic Scoring System (DIPSS) score ≥ Intermediate-1, an Eastern Cooperative Oncology Group score of 0─2, and did not receive prior JAK2 therapy or bromodomain and extra terminal motif inhibitors. Patients had to have at least 2 symptoms with a score ≥3 or a total score of ≥12, as measured by the Myelofibrosis Symptom Assessment Form (MFSAF) v4.0. Patients were excluded if they had splenic irradiation ≤6 months or splenectomy, accelerated/blast phase of myelofibrosis (>10% blasts in peripheral blood or bone marrow), prior therapy with a BH3 mimetic compound, stem cell transplantation, or platelet count <100 × 10^9^/L.

**Treatment continuation, discontinuation, and compliance.** Treatment continued until disease progression, unacceptable toxicity, or until other protocol-defined criteria for discontinuation were met (requiring alternative therapy for myelofibrosis, significant noncompliance, pregnancy or breastfeeding, withdrawal of patient consent, or any other medical reason the study investigator deemed appropriate). All patients were followed-up for safety 30 days post treatment discontinuation. Those who discontinued for reasons other than disease progression were followed for approximately 12 weeks until disease progression or initiation of another treatment for myelofibrosis.

**Key exploratory endpoints.** Key exploratory endpoints included: (i) Assessment of SVR_35_ at any time on study per modified IWG criteria; (ii) duration of SVR_35_ response; (iii) ≥50% reduction in palpable splenomegaly from baseline per modified IWG criteria^22,26^; (iv) TSS_50_ at any time on study by MFSAF v4.0; (v) change from baseline in TSS; (vi) duration of anemia response; (vii) overall survival (OS); (viii) progression-free survival (PFS); and (ix) assessment of fatigue and impacts using the Patient Reported Outcome Measurement Information System (PROMIS) Short Form (SF) v1.0 – Fatigue 7a and measures of health-related QoL, assessed using the European Organization for Research and Treatment of Cancer Quality of Life (EORTC QLQ-C30).

In an additional post hoc analysis, the proportion of patients achieving allele frequency reduction from baseline of ≥20% at week 24 was also summarized among patients with baseline and post-baseline of variant allele frequency (VAF) of driver gene mutations of *JAK2*, *CALR*, and *MPL* assessed by Next Generation Sequencing (Flagship Biosciences, Inc., Morrisville, NC, USA) of peripheral blood.

**Statistical analysis.** Analyses were conducted using SAS version 9.4 (SAS Institute, Inc, Cary, NC). Demographics, duration of exposure and safety for navitoclax and ruxolitinib, and changes in BMF grade were summarized using descriptive statistics. SVR_35_ and TSS_50_ were calculated as the proportion of patients achieving ≥35% SVR or ≥50% TSS reduction respectively at week 24, with corresponding 95% confidence intervals (CI) derived by the Clopper-Pearson method. Absolute and percent change from baseline of spleen volume were summarized with mean, standard deviation (SD), median, minimum, and maximum. A change of ≥10 to <20 points from baseline in EORTC QLQ-C30 QoL was considered moderate improvement in patient QoL; recommended minimum importance range for PROMIS SF v1.0 – Fatigue 7a was 3 to 5 points. Safety analyses included all patients receiving ≥1 dose of navitoclax.

## Supplemental FIG 1. Study design


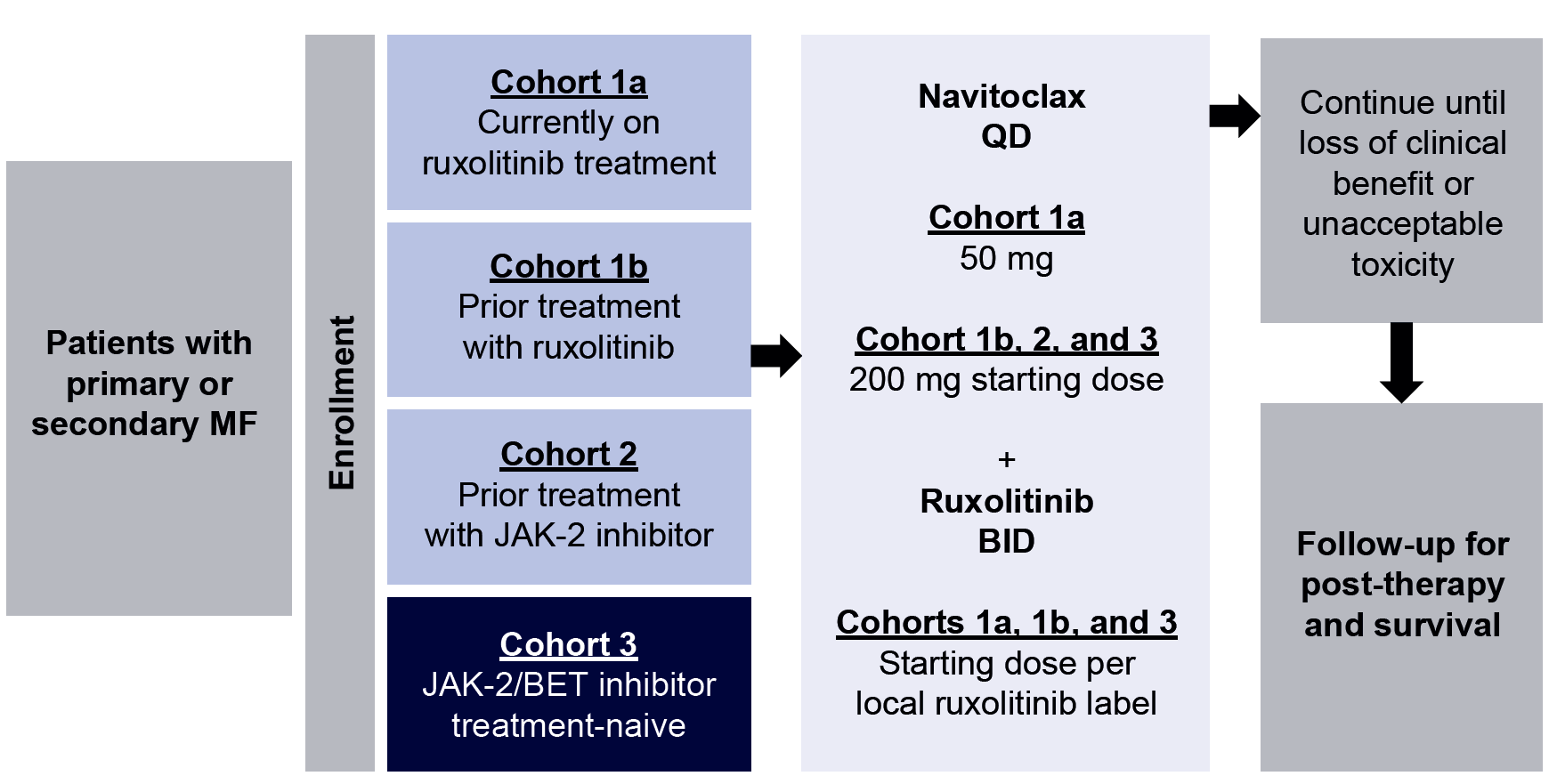


BET, bromodomain and extraterminal protein; BID, twice daily; JAK-2, Janus kinase-2; MF, myelofibrosis; QD, once daily.

## Supplemental FIG 2. Heat map showing baseline mutations in genes conferring high molecular risk


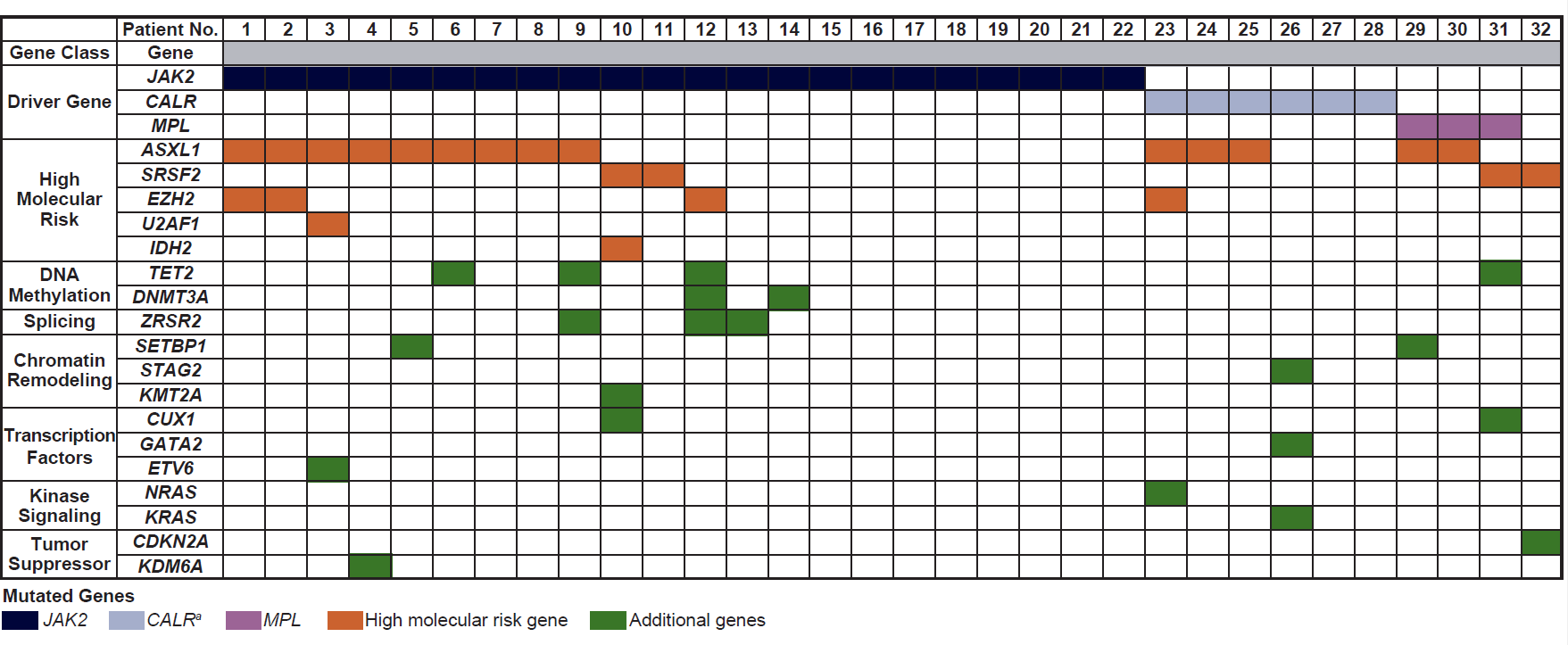


^a^Indicates all *CALR* type 1 mutations.

## Supplemental FIG 3. Patient disposition

Patients enrolled; N=32

Received ≥1 dose of navitoclax and ruxolitinib; N= 32

**Patients who remain on the study: n=0**

**Discontinuation of navitoclax: n (%), n=32 (100)**

- Adverse events: 6 (19)
- Progressive disease: 3 (9)
- Withdrew consent: 3 (9)
- Physician decision: 4 (13)
- Other: 16 (50)

**Discontinuation of ruxolitinib: n (%), n=32 (100)**

- Adverse events: 4 (13)
- Progressive disease: 3 (9)
- Withdrew consent: 2 (6)
- Physician decision: 4 (13)
- Other: 19 (59)

**Patients who discontinued study: n=32**

**Reasons for discontinuation: n (%)**

- Withdrew consent: 2 (6)
- Death: 10 (31)
  - TEAEs leading to death: 3 (9)
    - Cardiac disorder: 1 (3)
    - Respiratory failure: 1 (3)
    - Death: 1 (3)
- Other: 20 (63)
  - Study terminated by sponsor: 18 (56)
  - Patient became eligible and/or pursued transplant: 2 (6)

## Supplemental FIG 4. Swimmers plot of patient exposure to navitoclax and ruxolitinib by dose


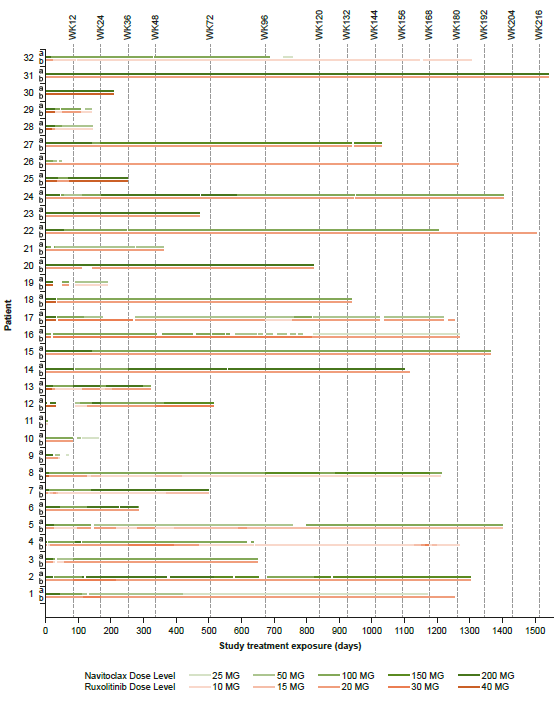


^a^Navitoclax total daily dose; ^b^Ruxolitinib total daily dose.

## Supplemental FIG 5. Absolute hematologic parameters over time. A) Platelets, B) hemoglobin, C) neutrophils


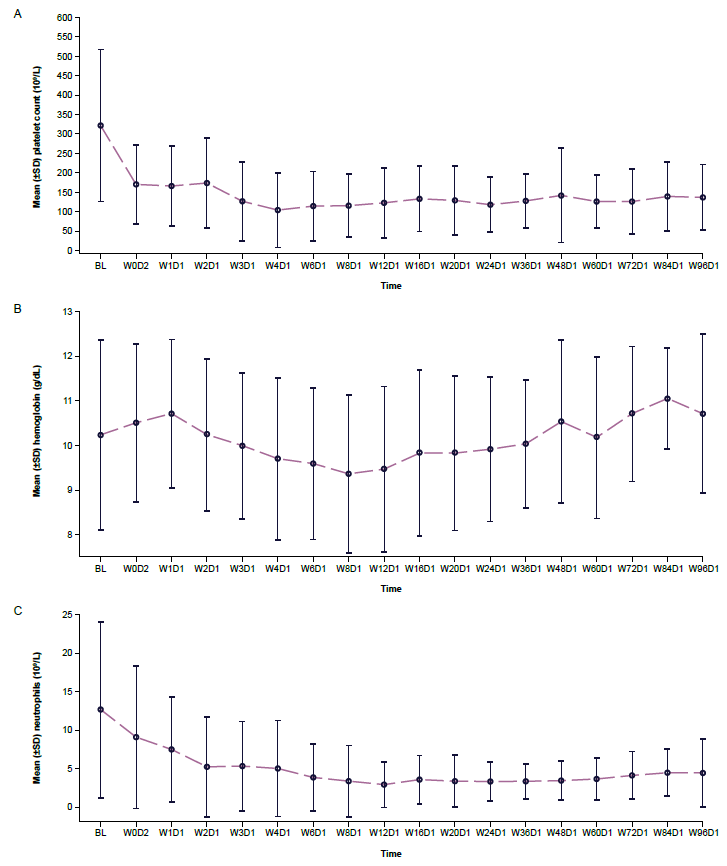


BL, baseline; D, day; SD, standard deviation; W, week.
